# Genome-Wide Association Study Identifies New Risk Loci for Progression of Schistosomiasis Among the Chinese Population

**DOI:** 10.3389/fcimb.2022.871545

**Published:** 2022-04-12

**Authors:** Miao Zhou, Chao Xue, Zhongdao Wu, Xiaoying Wu, Miaoxin Li

**Affiliations:** ^1^ Zhongshan School of Medicine, Sun Yat-Sen University, Guangzhou, China; ^2^ Key Laboratory of Tropical Disease Control, Ministry of Education, Sun Yat-Sen University, Guangzhou, China; ^3^ Provincial Engineering Technology Research Center for Biological Vector Control, Sun Yat-sen University, Guangzhou, China; ^4^ Department of Gastroenterology, The Third Affiliated Hospital of Sun Yat-Sen University, Guangzhou, China; ^5^ School of Public Health, Fudan University, Shanghai, China; ^6^ Center for Precision Medicine, Sun Yat-Sen University, Guangzhou, China; ^7^ State Key Laboratory of Brain and Cognitive Sciences, The University of Hong Kong, Hong Kong, Hong Kong SAR, China

**Keywords:** schistosomiasis, GWAS (genome-wide association study), *Schistosoma japonicum*, genetic susceptibility, hepatic fibrosis, portal hypertension, ascites

## Abstract

*Schistosoma japonicum* infections, which lead to local inflammatory responses to schistosome eggs trapped in host tissues, can result in long-term, severe complications. The development of schistosomiasis may result from a complex interaction between the pathogenic, environmental, and host genetic components. Notably, the genetic factors that influence the development of schistosomiasis complications are poorly understood. Here we performed a genome-wide association study on multiple schistosomiasis-related phenotypes of 637 unrelated schistosomiasis patients in the Chinese population. Among three indicators of liver damage, we identified two novel, genome-wide significant single-nucleotide polymorphisms (SNPs) rs34486793 (*P* = 1.415 × 10^-8^) and rs2008259 (*P* = 6.78 × 10^-8^) at locus 14q32.2 as well as a gene, *PMEPA1*, at 20q13.31 (index rs62205791, *P* = 6.52 × 10^-7^). These were significantly associated with serum levels of hyaluronic acid (HA). In addition, *RASIP1* and *MAMSTR* at 19q13.33 (index rs62132778, *P* = 1.72 × 10^-7^) were significantly associated with serum levels of aspartate aminotransferase (AST), and *TPM1* at 15q22.2 (index rs12442303, *P* = 4.39 × 10^-7^) was significantly associated with serum levels of albumin. In schistosomiasis clinical signs, *ITIH4* at 3p21.1 (index rs2239548) was associated with portal vein diameter (PVD) class, an indicator of portal hypertension, and *OGDHL* at 10q11.23 (index rs1258172) was related to ascites grade. We also detected an increased expression of these six genes in livers of mice with severe schistosomiasis. Summary data-based Mendelian randomization analyses indicated that *ITIH4*, *PMEPA1* and *MAMSTR* were pleiotropically associated with PVD class, HA and AST, respectively.

## Introduction

Schistosomiasis is a neglected tropical disease caused by parasitic flatworms (blood flukes) of the genus *Schistosoma*, with considerable morbidity and mortality in parts of the Middle East, South America, Southeast Asia and, particularly, sub-Saharan Africa (Burke et al., 2009; McManus et al., 2018). According to the World Health Organization (WHO), at least 230 million people worldwide are infected with *Schistosoma* species, with 700 million at risk (Colley et al., 2014; WHO, 2022). Although China has made remarkable achievements after conducting comprehensive prevention and control over the past six decades, schistosomiasis japonica caused by *Schistosoma japonicum* remained endemic in 240 counties in eight provinces, with 44 million people at risk. At the end of 2020, it was estimated that the total number of schistosomiasis patients in China was 83,179, with a cumulative total of 29,517 cases of advanced schistosomiasis (Zhang et al., 2021). Even if an infected person receives timely chemotherapy, the hepatic fibrosis (HF) may worsen and eventually develop into advanced schistosomiasis, which seriously affects the quality of life (Yepes et al., 2015; Kamdem et al., 2018; Kong et al., 2020). Moreover, it is impractical to change the residents’ occupation, making them still at high risk of acquiring infection and reinfection. Therefore, the task of eliminating schistosomiasis, especially advanced schistosomiasis, remains serious.

Adult schistosomes live within the mesenteric venules. After male-female worm paring, many eggs are not excreted but rather are deposited permanently in the intestines or liver along with the bloodstream (Hayashi, 2003; Gryseels et al., 2006; Burke et al., 2009). Mature eggs secrete soluble egg antigens, which are taken up by antigen-presenting cells such as macrophages and neutrophils, forming egg granulomas and eventually leading to acute and then chronic schistosomiasis colitis and pathognomonic periportal fibrosis (Elbaz and Esmat, 2013). This fibrosis leads to progressive occlusion of the portal veins, leading to the development of portal hypertension. B-ultrasound can detect the widening of the diameter of the hepatic portal vein (>13mm). Severe hepatic fibrosis and portal hypertension can further lead to infiltration of intravascular fluid into the abdominal cavity, resulting in ascites (Hayashi, 2003; Burke et al., 2009). Portal hypertension due to HF is a major cause of disease morbidity and mortality (Olveda et al., 2014). Unlike other liver diseases, periportal fibrosis caused by schistosomiasis preserves its hepatocyte function in the absence of co-infection with the liver virus, that is, in the early or chronic stages of schistosomiasis, patients have normal liver function biochemical indicators. In Brazil, subjects of African origin may be heavily infected but display little HF (Bina et al., 1978). This evidence suggests that, in addition to the frequency of infections, living environment, health status, lifestyle, and other factors, a host’s genetic factors may also affect the disease process and the degree of damage. It is worth noting that what genetic factors can contribute to or predispose one to these complications have yet to be fully understood.

Previous studies on the genetic mechanism of schistosomiasis have been based on early linkage examinations to identify major quantitative trait locus (QTL) loci. A comprehensive review summarized SNPs associated with schistosomiasis infection and pathology-related phenotypes from candidate gene studies (Mewamba et al., 2021). Among these, a study identified two SNPs in *IL5* with a significant association with susceptibility to *S. japonicum* symptomatic infection (Ellis et al., 2007). There are five genes for which an effect has only been reported for fibrosis and not for *S. japonicum* infection (*HSPA5*, *IL22RA2*, *MAPKAP1*, *mTOR* and *AKT2*). *IL22RA2* and *MAPKAP1* are associated with fibrosis in more than one population (Zhu et al., 2014; Sertorio et al., 2015; Xiao et al., 2020). *IL13* has been associated with both fibrosis and the intensity of *S. japonicum* infection. An SNP in major histocompatibility complex class I chain-related A (*MICA*) is associated with HF in a Han Chinese population (Gong et al., 2012). Polymorphisms of the *CTGF* gene, localized at 6q23, are associated with severe HF in Sudanese, Brazilian, and Chinese individuals infected with schistosomes (Dessein et al., 2009). Various polymorphisms in the transforming growth factor-beta (TGF-β) signaling pathways are associated with severe hepatic disease, including *ALK1* and *ALK5* (Vorstenbosch et al., 2017; Dessein et al., 2020). Advanced schistosomiasis is associated with elevated sST2 levels, and *ST2* genetic variants are associated with sST2 levels in patients infected with *S. japonicum* (Long et al., 2017). However, all such studies have been based on prior knowledge and have analyzed specific gene regions *via* case-control experiments. Most studies on susceptibility to schistosomiasis have focused on the immune genetic mechanism of HF. Few have applied multiple testing corrections. Thus, the existing data do not fully clarify the genetic factors of schistosomiasis and its complications. In this study, to obtain susceptibility loci and genes associated with the severity of schistosomiasis as much as possible, we performed a genome-wide association study (GWAS) to delineate host genetic factors contributing to the severity of schistosomiasis and its complications among the Chinese population.

## Materials and Methods

### Participants and Data Collection

To detect novel loci conferring susceptibility to schistosomiasis severity, we recruited 984 individuals with a history of schistosomiasis from nine counties around Poyang Lake in Jiangxi Province of China, an epidemic area of schistosomiasis. The participants were from the epidemiological survey of schistosomiasis japonicum in Jiangxi Province from 2015 to 2017. Nine counties were randomly selected for investigation in 32 counties within the scope of medical assistance for late-stage schistosomiasis japonica in Jiangxi Province. According to the “Diagnostic Criteria for Schistosomiasis” issued by the Ministry of Health of the People’s Republic of China in 2006 and “The Prevention and Control of Schistosomiasis and Soil-Transmitted Helminthiasis” reported by the WHO, participants were selected by professional laboratory and pathologists after evaluation. The inclusion criteria of the study subjects included: (1) a history of long-term or repeated exposure to infected water; (2) a clear history of treatment for schistosomiasis japonicum, or fecal examination found worm eggs or miracidia, or rectal biopsy found schistosomiasis eggs, or positive serum immunological test for long-term; (3) Exclude the subjects who self-reported a history of schistosomiasis, but were negative in serum immunological examination and had no information of B-ultrasound liver fibrosis. Ten milliliters of anticoagulated peripheral blood samples were collected on an empty stomach for breakfast from schistosomiasis patients who meet the above criteria. Serum samples were used to measure the biochemical indices, and blood cells were used to extract the genomic DNA.

Biological information was collected at the local schistosomiasis control station *via* face-to-face questionnaire interviews and physical examination. The physical examinations such as blood pressure, height, weight and waist circumference of the research subjects were repeated three times, and the average value was taken. The questionnaire contained sociodemographic characteristics (age, education level, occupation, and income level, e.g.), medical history of schistosomiasis, history of contact with infected water contact, and life behavior. The portal vein diameter (PVD), ascites, and related imaging markers were measured by abdominal ultrasonography. The detection of biochemical indicators was completed by Guangzhou Jinyu Medical Laboratory Center.

### Definition

Clinical classifications of schistosomiasis patients were made according to “Prevention and control of schistosomiasis and soil-transmitted helminthiasis” report of the WHO and the “WS 261-2006 diagnostic criteria for schistosomiasis”. The patient was placed in the right lateral decubitus, and the PVD was measured by a professional physician using B-ultrasound. The PVD ≥ 13 mm was considered widened (abnormal), and PVD < 13 mm was considered normal. “Age” is the actual age of the research object in the year under investigation. “Region” is the county where the research object is located. “Sex” 0=male, 1=female. “Smoking” 0=no smoking behavior, 1=yes. “Score of exposure to epidemic water” 1=rare exposure, 2=exposed several times a year, 3=every Monthly exposure, 4=every week. “Course of the disease” is the time from the first diagnosis to the investigation year; “Alcohol drinking” 0=no, 1=yes. The unit of AST is U/L. The unit of ALB is g/L. The unit of HA is ng/mL. The unit of total protein is g/L. Body mass index (BMI) was weight (kg) divided by the square of height (m), and the unit of BMI was kg/m^2^. Obesity was defined as BMI ≥ 30 kg/m2, “Obesity” 0=no, 1=yes. Central obesity refers to waist circumference ≥ 85 cm in men and ≥ 80 cm in women, and “central obesity” 0 = no, 1 = yes. The diagnosis of hypertension adopts the standard recommended by the 2005 Chinese Hypertension Treatment Guidelines: systolic blood pressure ≥140 mmHg, diastolic blood pressure ≥90 mmHg, “hypertension” 0=no, 1=yes. The grade of ascites is assessed by professional doctors by abdominal B-ultrasound. According to the report of the International Ascites Club consensus meeting, ascites is divided into three grades: grade 1 ascites: mild ascites, which can only be detected by ultrasound or computerized tomography (CT); grade 2 ascites: moderate ascites, manifested as moderately symmetrical abdominal distention; grade 3 ascites: massive or severe ascites, accompanied by obvious abdominal distention.

The samples were divided into advanced (or severe) and chronic (or moderate) categories based on the PVD classes roughly. Then, to measure the association between schistosomiasis-related clinical complications and severity, the Chi-square test (for categorical traits) or Student’s t-test (for continuous traits) was performed to compare the differences of clinical characteristics between the PVD normal (n=460) and abnormal (n=177).

### Genotyping, Quality Control, and Imputation

Based on automated nucleic acid purification platform (BioTeke, AU1001, Beijing, China), genomic DNA was extracted from the 200 μl of frozen blood cells using the magnetic bead-based method for concentrating DNA (The Genomic DNA Magnetic Beads Kit, AU18016, BioTeke, Beijing, China). Then the Illumina Infinium Asian Screening Array was used to genotype 659,184 SNPs. After stringent quality filtering, a total of 497,495 SNPs from 900 samples were ultimately retained. Next, to extend the coverage to the genomic region, autosomal SNPs that passed strict quality checks were used to impute genotypes of SNPs across the chromosomes for all subjects, and 6,237,938 SNPs were genotyped. Finally, after quality controls and relatedness removal, a total of 4,523,335 remained, representing 637 independent individuals.

We performed strict quality control on samples and SNPs to guarantee robust association analyses. Briefly, samples were excluded if they (a) had an overall genotyping rate of <90%; (b) showed unexpected duplicates or relatives (PI_HAT > 0.05), or (c) were identified as outliers. PCA was used to detect the outliers using Genome-wide Complex Trait Analysis software (GCTA; version 1.92.2). Twenty principal components were calculated, and the first four ancestry principal components explained 60% variation. In addition, SNPs were excluded if (a) the call rate of <90%; (b) minor allele frequency (MAF) was <0.05; (c) did not map to autosomal chromosomes; and (d) if the *P*-value was less than 1.0 × 10^-5^ in Hardy-Weinberg equilibrium tests.

Following the prephasing of genotypes with Shape-IT (Delaneau et al., 2012) v2, we imputed genetic markers from the 1000 Genomes Project reference panel (phase III integrated release; http://1000genomes.org) using IMPUTE2 (Howie et al., 2009) v2.3.0. We used the information *r^2^* from IMPUTE2 as a QC parameter, which estimates the correlation between imputed and true genotypes. Genetic markers with an imputation *r^2^* < 0.4 were removed.

### GWAS Analysis

We used PLINK (Purcell et al., 2007) v1.9 (www.cog-genomics.org/plink/1.9/) to analyze the associations between genome-wide SNPs and relevant indicators of the risk for schistosomiasis. Logistic regression models were used for disease traits (PVD), and linear regression was used for quantitative features (HA, AST, albumin, ascites grade). For the selection of PCA covariates, we used EIGENSTRAT (Price et al., 2006) to calculate the number of significant principal components, and selected the first three significant principal components based on a *P* value of 0.05. We used non-genetic factors (characteristics in **Table 1**) as independent variables to construct linear regression models for serum HA, AST, serum albumin and ascites grade and logistic regression models for the PVD class, respectively. And then, stepwise regression was used to screen the optimal set of covariates for each phenotype. For the HA, association tests were adjusted for region, age, history of infected water contact, and the first three ancestry PCs. For the AST, association tests were adjusted for region, age, and the first three ancestry PCs. For albumin, association tests were adjusted for the area, sex, age, history of infected water contact, and the first three ancestry PCs. For ascites, association tests were adjusted for region, history of infected water contact and the first three ancestry PCs. For PVD, association tests were adjusted for the area, sex, age, smoking, course of the disease, and the first three ancestry PCs. We modeled data from each genetic marker using additive dosages to account for imputation uncertainty. The effective number of independent SNPs was calculated in Genetic type 1 Error Calculator (GEC) (Li et al., 2012) (http://pmglab.top/gec/). The effective number of independent tests for all the 3,949,613 valid variants is 514484.83 (13.03%). The genome-wide significant *P* value cutoff by Bonferroni correction was approximately 1 × 10^−7^, and the suggestive *P* value was 1 × 10^−5^.

**Table 1:** Statistical test to compare the statistical discrepancy of clinical characteristics between schistosomiasis patients at different stages.

Category	Stat. Method	Stat. Val.	P
**Characteristics**			
Age	T-test	3.476	0.001
Sex	Chi square test	23.909	1.01E-06
Smoking	Chi square test	2.677	0.102
Score of exposure to epidemic water	Chi square test	50.141	7.46E-11
Course of the disease	T-test	2.735	0.006
Alcohol drinking	Chi square test	0.990	0.320
**Symptoms**			
Aspartate transaminase	T-test	4.799	1.84E-06
Serum Albumin	T-test	-7.356	4.00E-13
Hyaluronic Acid	T-test	8.621	2.63E-17
Total protein	T-test	0.083	0.934
BMI	T-test	0.073	0.941
Obesity	Chi square test	3.808	0.149
Central obesity	Chi square test	0.017	0.896
Hypertension	Chi square test	0.933	0.334
Ascites Grade	Chi square test	65.719	3.52E-14

### Genomic Risk Locus Definition

Genomic risk loci were defined using FUMA (Watanabe et al., 2017), an online platform for functional mapping and annotation of genetic variants. We first identified significant independent SNPs with a suggested significant two-tailed *P* value (*P* < 1 × 10^−5^) and which were independent of each other at *r^2^* < 0.6. Lead SNPs or index SNPs further represented these SNPs, a subset of the significant independent SNPs, with the most significant *P* value in approximate linkage equilibrium. Next, we defined independent genomic risk loci by merging any physically overlapping lead SNPs (LD blocks < 250 kb apart). The borders of the genomic risk loci were determined by identifying all SNPs in LD (*r^2^* ≥ 0.6) with one of the significant independent SNPs in the locus; the region containing all of these candidate SNPs was considered a single independent genomic risk locus. All LD information was calculated using the 1000 genome phase III EAS genotype data as a reference.

### Functional Annotation of SNPs

Functional annotation of the SNPs implicated in this study was performed using FUMA. We selected all candidate SNPs in genomic risk loci having an *r^2^* ≥ 0.6 with one of the significant independent SNPs, a *P-*value 1 × 10^−5^, and a MAF > 0.05. Predicted functions of these SNPs were obtained by matching their chromosomes, base-pair positions, and reference and alternate alleles to databases containing known functional annotations, including CADD, RegulomeDB. CADD scores predict how harmful the effect of an SNP is likely to be for non-coding gene/protein structure/function, with higher scores referring to higher deleteriousness. A CADD score above 12.37 is the threshold for potential pathogenicity. The RegulomeDB score is a categorical score based on eQTLs and chromatin marks, ranging from 1a to 7, with lower scores indicating an increased likelihood of having a regulatory function. The chromatin state represents the accessibility of genomic regions (every 200 bp). A lower state means higher accessibility, with states 1–7 referring to open chromatin states. We annotated the minimum chromatin state across tissues to SNPs.

### Gene Mapping

Significant loci obtained by GWAS were mapped to genes in FUMA using three strategies:

(1) Positional mapping of SNPs to genes based on physical distance (within a 10 kb window) from known protein-coding genes in the human reference assembly (GRCh37/hg19).
(2) eQTL mapping of SNPs to genes with which they showed a significant eQTL association (i.e., allelic variation at the SNP is associated with the expression level of that gene). eQTL mapping uses information from 54 tissue types in three data repositories (GTEx, Blood eQTL browser, BIOS QTL browser) and is based on cis-eQTLs that can map SNPs to genes up to 1 Mb away. We used a false discovery rate (FDR) of 0.05 to define significant eQTL associations.
(3) CI mapping of SNPs to genes when there are DNA–DNA interactions between the SNP region and a gene region. CI mapping can involve long-range interactions, as it does not have a distance boundary, and an interacting region can span multiple genes. We used an FDR of 1 × 10^−5^ to define significant interactions, based on previous recommendations modified to account for the differences in the cell lines used here.

### Summary Mendelian Randomization

We conducted the SMR analysis with GTEx.v8 eQTL as the instrumental variables, gene expression as the exposure, and each of the five traits as the outcome. The analysis was conducted according to the method as implemented in the SMR & HEIDI methods and software tools (Zhu et al., 2016). The tool was originally developed to test for pleiotropic associations between the expression levels of a gene and a complex trait of interest. The methodology can be interpreted as an analysis to test if the effect size of an SNP on a phenotype is mediated by gene expression. We also conducted the heterogeneity in dependent instruments (HEIDI) test to evaluate the existence of linkages in the observed associations. Rejection of the null hypothesis (*P*_HEIDI_ < 0.1) indicates that the observed association could be due to two distinct genetic variants in high linkage disequilibrium with each other.

### qRT-PCR

The expression of target mRNA was determined using the SYBR Green Master Mix kit (Takara, Japan). GAPDH was used as an internal control, and the fold change was calculated by the 2^−ΔΔCT^ method. For additional details, see supplementary information.

## Results

### Association Between Schistosomiasis-Related Clinical Symptoms and Severity

We first examined the association between schistosomiasis-related clinical symptoms and disease severity in 984 individuals with a history of schistosomiasis (including chronic and advanced stage) from nine counties around Poyang Lake in Jiangxi Province of China. We found that three biochemical indicators of liver injury including serum levels of aspartate transaminase (AST, *P*_T-test_ = 1.84 × 10^-6^), serum levels of albumin (*P*_T-test_ = 4 × 10^-13^), serum levels of hyaluronic acid (HA, *P*_T-test_ = 2.62 × 10^-17^) and ascites grade (*Px* ^2^ = 3.52 × 10^-14^) showed significant differences between the PVD normal (n=745) and abnormal (n=239), which means that these indicators were related to the progression of schistosomiasis (**Table 1**). Severe schistosomiasis patients present with abnormal portal vein diameter (PVD) ≥ 13 mm, elevated AST and HA levels, lower levels of albumin, and higher ascites grade. Serious cases were more frequently male (*Px* ^2^ = 1.01 × 10^-6^). A higher frequency of exposure to epidemic water and longer disease duration is positively correlated with more severe schistosomiasis disease (*P* = 7.46 × 10^-11^ and 0.0064, respectively); see detail in **Figure S1**.

### Genome-Wide Association Analysis of Five Schistosomiasis-Related Clinical Symptoms

**Figure 1:**
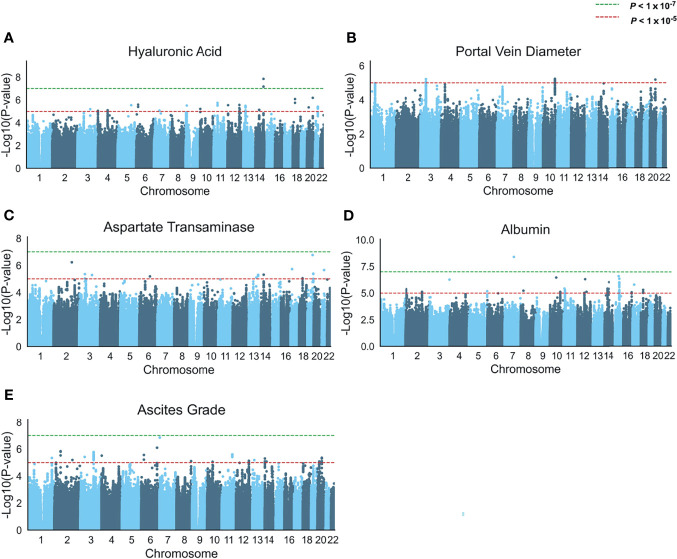
Genome-wide association analysis of five schistosomiasis-related indicators. **(A–E)** Manhattan plots of the association statistics from five GWAS analyses (controlled for potential population stratification, n = 637) **(A)** Serum levels of hyaluronic acid **(B)** Portal vein diameter (abnormal n= 177 and normal n=460) in a logistic regression model, **(C)** Serum levels of aspartate transaminase, **(D)** Serum levels of albumin, and **(E)** Ascites grade. The y axis shows the negative log10-transformed SNP two-sided *P* value from a linear or logistic regression model, and the x-axis is the base-pair position of the SNPs on each chromosome. The green dashed line indicates the genome-wide significant *P* value (*P* < 1 × 10^-7^) cutoff by Bonferroni correction, the red dashed line indicates a suggestive threshold (*P* < 1 ×  10^-5^).

We then carried out a GWAS on the five above-mentioned schistosomiasis-related clinical symptoms (HA, AST, albumin, PVD, and ascites grade) among 637 non-related individuals after excluding 263 related subjects (**Figure 1**). A quantile-quantile (Q–Q) plot suggested no overall inflation of the genome-wide statistical results (λ_HA_ = 0.983, λ_PVD_ =1.022,λ_AST_ = 1.017, λ_Albumin_ = 1.008, λ_Ascites_ = 1.001; **Figure S2**). However, only HA and albumin had whole-genome-level significantly (*P* < 1 × 10^-7^) associated SNPs. In addition, there were several loci with suggestively significant *P* values for the other three clinical symptoms (*P* < 1 × 10^-5^). Most of these associated loci in these five traits were intronic or intergenic (**Figure S3**).

### Genome-Wide Association Analysis for Schistosomiasis Liver Damage

#### Chromosome 14q32.2 Locus Is Associated With Hyaluronic Acid Levels

**Figure 2:**
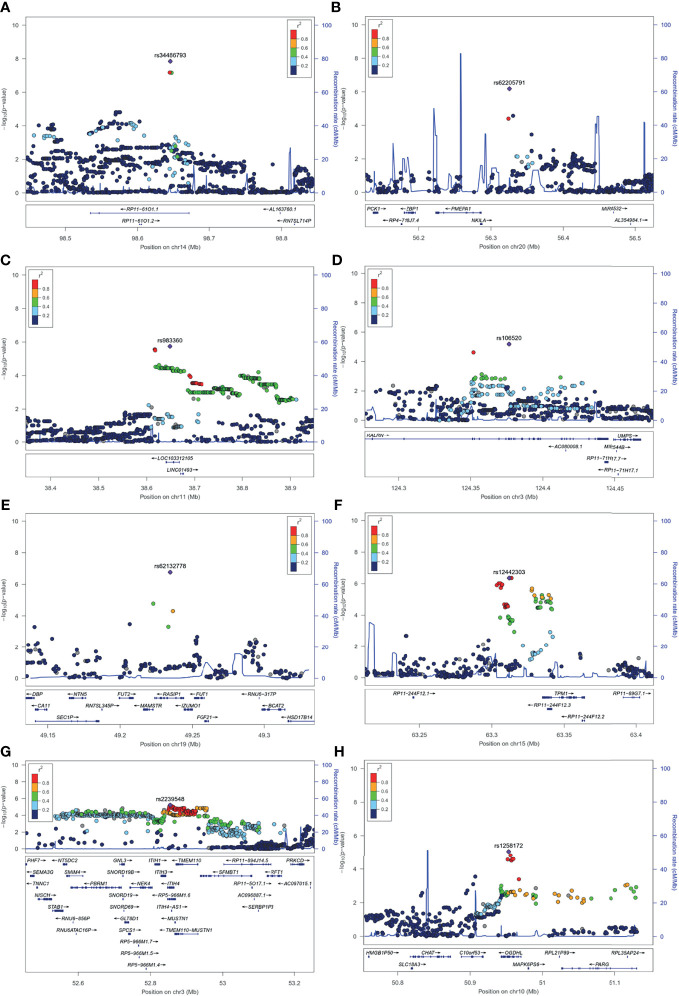
Regional plots for novel association loci. The x-axis represents the chromosomal position, and the y axis is the –log10 of the *P* value of association. The index SNPs are shown as purple diamonds. Colors indicate the LD level with the index SNP. Red signifies *r^2^* ≥ 0.8, with orange 0.6 ≤ *r^2^* < 0.8, green 0.4 ≤ *r^2^* < 0.6, light blue 0.2 ≤ *r^2^* < 0.4, and dark blue *r^2^* < 0.2. Blue lines represent recombination hotspots estimated in the East Asian population (from the 1000 Genomes Project, November, 2014). Genomic positions are based on human genome assembly hg19. The plot shows the names and locations of the genes; the transcribed strand is indicated with an arrow. Index SNPs **(A)** rs34486793 at 14q32.2. **(B)** rs62205791 at 20q13.31. **(C)** rs983360 at 11p12. **(D)** rs106520 at 3q21.1. **(E)** rs62132778 at 19q13.33. **(F)** rs12442303 at 15q22.2. **(G)** rs2239548 at 3p21.1. **(H)** rs1258172 at 10q11.23.

HA is an essential component of the extracellular matrix (ECM) (Parés et al., 1996). Serum levels of HA are a good indicator to reflect the degree of hepatic endothelial cell damage and HF. Consistent with other research findings, serum levels of HA are positively correlated with the severity of schistosomiasis (**Table 1** and **Figure S1**). Eighteen genomic risk loci (*P* < 1 × 10^−5^) were obtained in the GWAS of HA, including 20 significant independent SNPs and 18 lead SNPs (**Table S1**). We identified one locus associated with HA that reached the genome-wide significance threshold of *P* = 1 × 10^-7^, i.e., the chromosome 14q32.2 (insertion and deletion mutation rs34486793, *P* = 1.415 × 10^-8^ and another independent significant SNP rs2008259, *P* = 6.78 × 10^-8^; **Table 2** and **Figure 2** **A**). This locus located at lincRNA *RP11-61O1.1*, with a high imputation quality (IMPUTE2 info score > 0.97), suggests that the association between this locus and HA content is unlikely to be a false positive caused by imputation errors. The SNP rs34486793 showed a highly observed probability of a deleterious effect (CADD score = 18.75) and was also significantly associated with the ascites phenotype (*P* = 7.73 × 10^-5^, **Table 2** and **Table S2**). We also assessed whether this locus is significantly associated with other traits or diseases. We focused on the SNPs with *r^2^* ≥ 0.8 concerning the SNP rs34486793 and rs2008259. All of them were associated with eosinophil counts in the European population (**Table S4**) (Astle et al., 2016).

**Table 2:** Association results in five schistosomiasis clinical symptoms.

Phenotype	Loci	SNP(Minor/Major allele)	MAF^e^	Beta^f^(95% CI)	OR ^g^(95% CI)	*P* Value^h^	Info^i^	Beta in other phenotypes(95% CI)	*P* value in other phenotypes(< 1 × 10^-4)^
HA ^a^	14q32.2	rs34486793(GT/G)	0.43	0.22(0.14, 0.29)		1.42 × 10^-8^	0.968	0.16(0.08, 0.24)	*P*_(AG)_ = 7.73 × 10^-5^
		rs2008259(T/C)	0.47	-0.21(-0.28, -0.13)		6.78 × 10^-8^	0.974		
	20q13.31	rs62205791(G/A)	0.05	0.19(0.11, 0.26)		6.52 × 10^-7^	1		
		rs62205790(C/T)	0.05	0.16(0.08, 0.23)		3.93 × 10^-5^	0.94		
	11p12	rs983360(T/G)	0.13	0.18(0.11, 0.26)		1.82 × 10^-6^	0.944	-0.14(-0.21, -0.07)	*P*_(Albumin)_ = 7.83 × 10^-5^
	3q21.1	rs106520(G/A)	0.23	0.17(0.10, 0.25)		6.49 × 10^-6^	0.97	0.17(0.09, 0.25)	*P*_(AG)_ = 2.61 × 10^-5^
AST ^b^	19q13.33	rs62132778(C/G)	0.24	0.20(0.12, 0.27)		1.72 × 10^-7^	0.996		
Albumin	15q22.2	rs12442303(T/C)	0.44	0.18(0.11, 0.25)		4.39 × 10^-7^	0.966		
PVD ^c^	3p21.1	rs2239548(C/A)	0.39/0.26 ^j^		0.52(0.40, 0.69)	6.06 × 10^-6k^	0.995		
AG ^d^	10q11.23	rs1258172(T/C)	0.06	0.17(0.10, 0.25)		8.79 × 10^-6^	0.989	-0.14(-0.21, -0.07)	*P*_(Albumin)_ = 5.72 × 10^-5^

^a^HA, hyaluronic acid.

^b^AST, aspartate transaminase.

^c^PVD, portal vein diameter.

^d^AG, ascites grade; SNP, single-nucleotide polymorphism.

^e^MAF, minor allele frequency.

^f^Beta, Regression coefficient. For the additive effects of SNPs, the direction of the regression coefficient represents the effect of each extra minor allele; 95% CI, 95% confidence interval.

^g^OR, Estimated odds ratio (for minor allele).

^h^P value, P value of GWAS analysis.

^i^Info, IMPUTE2 imputation info score.

^j^MAF in control (PVD<13mm) and case (PVD≥13mm), respectively.

^k^P value based on logistic regression (PVD abnormal n=177 and normal n=460). After quality controls, the cohort includes 637 independent individuals. In HA, AST, Albumin and AF phenotypes, the associations were calculated using linear regression models adjusted for variables that are significant in stepwise regression and top three principal components. The associations of PVD phenotype were calculated using logistic regression models adjusted for variables that are significant in stepwise regression and top three principal components. ORs and 95% CIs are shown with respect to the minor alleles.

#### Chromosome 20q13.31 Locus Is Associated With Hyaluronic Acid Levels

**Figure 3:**
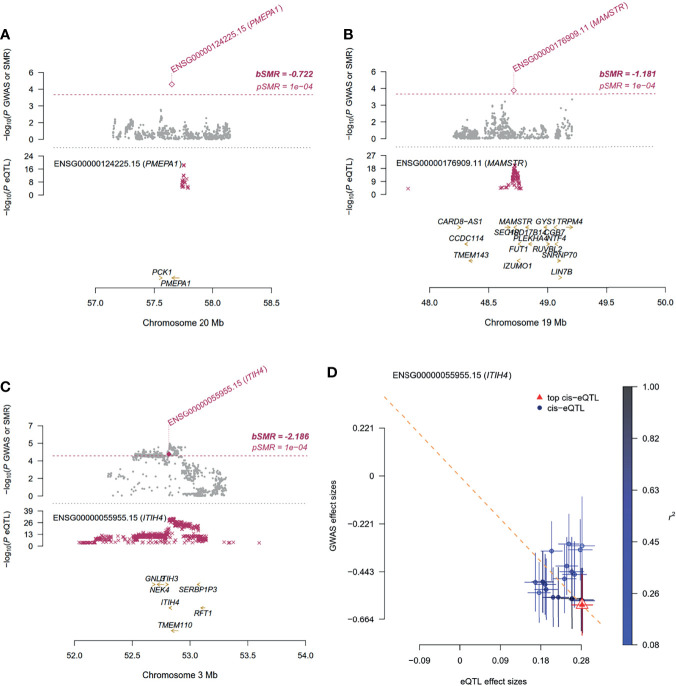
Summary-data-based Mendelian Randomization (SMR) analysis using gene expression quantitative trait loci (eQTL) implicates the role of associated genes in schistosomiasis. For **(A–C)** Top plot, gray dots represent the *P* values for SNPs from the GWAS analysis for associated genes **(A)** *PMEPA1*, **(B)** *MAMSTR* and **(C)** *ITIH4*, diamonds represent the *P* values for probes from the SMR test; Bottom plot, the eQTL *P* values of SNPs from GTEx v8 for the probes tagging associated genes. The top and bottom plots include all the SNPs available in the region in the GWAS and eQTL summary data, respectively, rather than only the SNPs common to both data sets. Highlighted in red is the gene that passed the SMR and HEIDI tests. **(D)** Effect sizes of SNPs (used for the HEIDI test) from GWAS plotted against those for SNPs from the GTEx v8 eQTL studies. The orange dashed lines represent the estimate of *b_xy_* at the top cis-eQTL (rather than the regression line). Error bars are the standard errors of SNP effects.

The association signal at locus 20q13.31 comprised three genes (i.e., *PMEPA1*, *NKILA* and *MIR4532*) (**Figure 2** **B**). The lead SNP rs62205791 (standard beta for the G allele = 0.1863, *P* = 6.52 × 10^-7^; **Table 2**) was located in an intergenic region of it. The rs62205791 expression quantitative trait loci (eQTL) mapped to seven genes in nine tissues according to the QTLbase (Zheng et al., 2019), and the risk allele G was significantly associated with lower expression of *PMEPA1* in the European population (the most significant *P* = 3.06 × 10^-16^, **Table S5**). Annotation results showed that rs62205790 highly linked with lead SNP (Linkage disequilibrium, LD *r^2^* = 0.96) had the highest functional score at this locus (CADD score = 21.6; **Table S2**) in FUMA. We applied the summary data-based Mendelian randomization (SMR) method to integrate GWAS summarized data and eQTL data to identify genes associated with HA levels. Five genes were also mapped by FUMA (**Figure S4** **A** and **Table S3**). It should be noted that the gene *PMEPA1* (*β*_SMR_ = -0.722, *P*_SMR_ = 1.15 × 10^-5^) was the most significant pleiotropically/potentially causal gene associated with HA (**Figure 3** **A** and **Table S6**). These data suggest that decreased gene expression of *PMEPA1* at 20q13.31 is linked to increased risk of elevated serum levels of HA in schistosomiasis patients.

Apart from this, GWAS of HA indicated that the lead SNP rs983360 (*P* = 1.82 × 10^-6^, **Table 2** and **Figure 2** **C**) located in the intronic region of ncRNA *RP11-277K23.1* at 11p12 and the lead SNP rs106520 (*P* = 6.49 × 10^-6^, **Table 2** and **Figure 2D**) located in the intronic of *KALRN* at 3q21.1 showed a significant association in the serum albumin phenotype (*P* = 7.83 × 10^-5^) and the ascites phenotype (*P* = 2.61 × 10^-5^; **Table 2**), respectively. In addition, eQTL and chromatin interaction (CI) analyses mapped several genes (including *IL3*, *IL5*, *1L13*, *IL4*, *IRF1* and *CSF2*) at the 5q31.1 region in the HA phenotype (**Figure S5A**). Numerous mutations within these genes at 5q31.1 are associated with eosinophil count, body height (Astle et al., 2016), fibrinogen levels and inflammatory bowel disease (Liu et al., 2015). It has been reported that the 5q31-q33 region is the locus that controls the intensity of *S. mansoni* infection (Marquet et al., 1996), and the polymorphisms of *IL4*, *IL5* and others are associated with HF in schistosome-infected subjects (Dessein et al., 2020).

#### Chromosome 19q13.33 Locus Is Associated With Aspartate Transaminase Levels

Serum levels of AST are an important indicator in liver function testing. The top SNP in GWAS of AST was rs62132778 (beta for the C allele = 0.1961, *P* = 1.72 × 10^-7^, **Table 2** and **Figure 2E**; **Table S7**), located in the intronic region of gene *RASIP1* at 19q13.33. The risk allele C of rs62132778 was significantly associated with lower expression of *RASIP1* and *MAMSTR* in fibroblast tissue in the European population (*P* = 2.84 × 10^-6^ and 6.37 × 10^-7^, **Table S8**). We also applied the SMR method to identify genes associated with AST. Interestingly, *MAMSTR* (top SNP rs62132778, *β*_SMR_= -1.181, *P*_SMR_= 4.83 × 10^-5^) showed the most significant pleiotropic association with AST (**Figure 3B** and **Table S9**). This suggests that rs62132778 at the 19q13.33 locus might be a causal variant, and decreased gene expression of *MAMSTR* is related to increased risk of elevated levels of AST in schistosomiasis patients.

#### Chromosome 15q22.2 Locus Is Associated With Serum Levels of Albumin

Albumin is produced in the liver and makes up approximately 60% of serum proteins (Sheinenzon et al., 2021). Serum levels of albumin are an important indicator of liver damage, which were significantly lower in patients with severe schistosomiasis (**Table 1** and **Figure S1I**). The lead SNP rs12442303 (*P* = 4.39 × 10^-7^, **Table 2** and **Figure 2F**) mapped to an intergenic region of the 15q22.2 flanking gene *TPM1*. RegulomeDB score = 1f indicates that rs12442303 was an eQTL located at transcription factor binding sites or DNase I hypersensitive sites (**Table S10**). The risk allele T of rs12442303 is significantly associated with lower expression of *TPM1* in blood and immune cell (the most significant *P* = 3.27 × 10^−310^; **Table S11**). According to the STRING v11.0 database (Szklarczyk et al., 2018), *TPM1* is co-expressed with the *ACTA2* gene encoding α-smooth muscle actin (α-SMA), which correlates with fibrosis degree (**Figure S6**). Previous research has demonstrated that the microRNA miR29c alleviates renal fibrosis *via* TPM1-mediated suppression of the Wnt/β-catenin pathway (Huang et al., 2020). Therefore, mutations in *TPM1* at 15q22.2 might play a potential role in developing schistosomiasis liver injury.

### Chromosome 3p21.1 Locus Is Associated With Portal Vein Diameter

PVD is considered an indicator of portal hypertension. PVD ≥ 13 mm suggests possible portal hypertension (Schabel et al., 1980). Among 637 independent samples, PVD normal (n=460) and abnormal (n=177). The lead SNP rs2239548 (Odds ratio for the C allele = 0.5242, *P* = 6.06 × 10^-6^; **Table 2** and **Figure 2G**) was located in an intronic region of *ITIH4* at 3p21.1. The allele C of rs2239548 is significantly associated with higher expression of *ITIH4* in the whole blood tissues, adipose tissue, arteries, and skeletal muscle (the most significant *P*_eQTL_ = 2.11 × 10^−21^; **Table S14**). We applied the SMR method to identify PVD-associated genes, of which seven were also mapped by FUMA (**Figure S4D**). SMR predicted a linear causal effect of cis-eQTL in *ITIH4* on PVD (*β*_SMR_ = -2.1863, *P*_SMR_ = 6.91 × 10^-5^, *P*_HEIDI_ = 0.45; **Figures 3C, D** and **Table S15**). Six genes (i.e., *RP11-894J14.2*, *NEK4*, *TMEM110*, *MUSTN1*, *GNL3*, *NT5DC2*) at the 3p21.1 locus showed significant pleiotropic associations in SMR analysis (**Table S15**). In addition, *ITIH4*, *TMEM110*, and *MUSTN1* satisfied position mapping, eQTL mapping, and CI mapping in FUMA (**Figure S5B** and **Table S13**). CIs at this locus affect *ITIH4* gene expression regulation in the liver and spleen tissues (**Table S13**). These results indicated that the 3p21.1 locus might be potentially involved in the development of schistosomiasis, and *ITIH4* is most likely the potential candidate gene at this locus.

### Chromosome 10q11.23 Locus Is Associated With Ascites Grade

Ascites can be caused by a combination of hypoalbuminemia and portal hypertension (Gryseels et al., 2006). The presence of ascites indicates that schistosomiasis is under the decompensated phase. Lead SNP rs1258172 (*P* = 8.79 × 10^-6^, **Table 2** and **Figure 2H**) is located in an intronic region of *OGDHL* at 10q11.23. RegulomeDB score = 2b indicated an increased likelihood of regulatory function (**Table S16**). In addition, the locus also exhibited a significant association in the serum albumin phenotype (**Table 2**). eQTL analysis mapped SNP rs1258172 to *OGDHL* in colon, esophagus, and fibroblasts (the most significant *P* = 5.76 × 10^−49^; **Table S17**). We identified *OGDHL* with previously reported phenotypic (*P* < 5 × 10^-8^) associations in published GWASs, two SNPs in *OGDHL* showed significant association with perforation of the intestine (**Table S18**).

### Increased Expression Levels of Six Associated Genes in Severe Schistosomiasis

**Figure 4:**
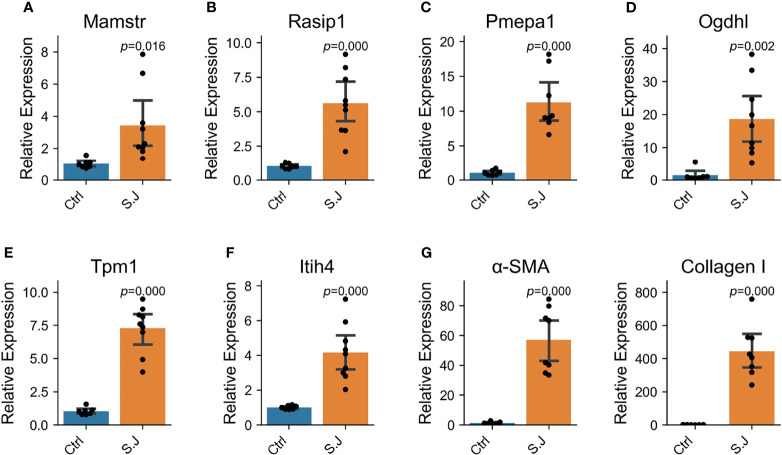
Six schistosomiasis-associated genes were up-regulated in the liver of mice with severe schistosomiasis. Expression levels of genes were determined by qRT-PCR in normal and *S. japonicum* infected liver (n = 8 per group). Bar and error bar represent mean and 95% confidence intervals, respectively. A two-tailed Student T-test was used for statistical analysis. **(A, B)** *Mamstr* and *Rasip1*, the associated genes of aspartate aminotransferase (AST). **(C)** *Pmepa*1, the associated gene of hyaluronic acid (HA). **(D)** *Ogdhl*, the associated gene of ascites grade. **(E)** *Tpm1*, the associated gene of albumin. **(F)** *Itih4*, the associated gene of Portal vein diameter (PVD). **(G)** α-SMA and Collagen I was the index reflecting the degree of liver fibrosis in mice with schistosomiasis.

Interestingly, significantly elevated expression of six above-mentioned associated genes (*Pmepa1*, *Mamstr*, *Rasip1*, *Tpm1*, *Itih4* and *Ogdhl*) in mice total liver tissues was also observed from the seventh week (*S. japonicum* spawn in large numbers) after infection (**Figure 4**). α-SMA is a marker of hepatic stellate cell activation, a pivotal event in the initiation and progression of HF, and collagen-1 reflects collagen deposition. Significant elevation of α-SMA and collagen-1 indicates severe HF (*P* < 0.000, **Figure 4G**). This result suggested that the six associated genes were involved in the regulation of the schistosomiasis process.

## Discussion

This study was the first to conduct GWAS analyses on genetic susceptibility to schistosomiasis complications severity among the Chinese population. We found four novel loci related to liver damage (14q32.2 [SNPs rs34486793 and rs2008259], 20q13.31 [SNP rs62205791], 19q13.33 [SNP rs62132778], and 15q22.2 [SNP rs12442303]), a locus associated with portal hypertension (3p21.1 [SNP rs2239548]), and a locus associated with ascites grade (10q11.23 [SNP rs1258172]).

The SNP rs34486793 and rs2008259 are located in lncRNA *RP11-61O1.1*. Although we have not yet found any literature describing the function of this gene, we found that mutations are related to eosinophil count, which makes it a biologically plausible causal factor for susceptibility to schistosomiasis severity. Eosinophils regulate the liver immune response and the response to tissue damage caused by *S. mansoni* infection in mice, which is necessary to balance liver damage and fibrosis (de Oliveira et al., 2022). In addition, we found that rs34486793 and rs106520 are significantly related in the ascites grade phenotype (*P* = 7.73 × 10^-5^ and 2.61 × 10^-5^, respectively), and rs983360 also had a significant correlation in the serum albumin phenotype (*P* = 7.83 × 10^-5^).

*PMEPA1* is also named *TMEPA1*. SMR analysis indicated that changes in *PMEPA1* expression might affect HA content and thus schistosomiasis severity (**Figure 3A**). Extensive studies have confirmed that the TGF-β signaling pathway plays an important role in the development of fibrosis (Ong et al., 2021). The expression of *PMEPA1* is induced by androgens and TGF-β, whose encoded protein suppresses the androgen receptor and TGF-β signaling pathway through interactions with Smad proteins (Xu et al., 2000; Watanabe et al., 2010). These also provide experimental biological evidence for causal genes indirectly. *PMEPA1* is upregulated in the liver of mice with severe schistosomiasis (**Figure 4C**). However, the mutations rs62205791 and rs62205790 are associated with lower expression of *PMEPA1*. The heterogeneity *P* value of these two SNPs was not calculated, so it is impossible to distinguish whether they are pleiotropic or LD effects. Therefore, these results indicate that the mutations in *PMEPA1* may underlie the risk of schistosomiasis HF and could be used to remind the patients with the G genotype for rs62205791 and the C genotype for rs62205790 of timely treatment.

The risk allele C of rs62132778 was positively correlated with AST content (**Table 2**) and showed an association with lower expression of *MAMSTR* and *RASIP1*. Previous studies on the function of *RASIP1* have mostly been conducted on lymphatic vessels (Liu et al., 2018). We found that *RASIP1* expression was upregulated in the liver of schistosome-infected mice (**Figure 4B**). Furthermore, *MAMSTR* was identified as the causal gene that affected the AST phenotype in SMR analysis. These results suggest that the genetic variants in *MAMSTR* and *RASIP1* may also be responsible for the risk of schistosomiasis liver disease.

The locus 3p21.1 (*ITIH4*, lead SNP rs2239548) is significantly associated with PVD. *ITIH4* is involved in the stabilization of the ECM and may play a role in liver development or regeneration. ITIH4 is a type II acute-phase protein involved in inflammatory responses to trauma, and its expression is altered in liver disease (Chandler et al., 2014). It has a significant correlation with the HF stage (*P* = 0.015) in children with chronic hepatitis C (Sira et al., 2014); our findings in the livers of schistosome-infected mice are consistent with that finding (**Figure 4F**). Notably, the effective C alleles of rs2239548 and *ITIH4* are highly protective against portal hypertension.

Schistosomiasis HF can continually develop even if the chronically infected patient is treated with praziquantel promptly. In decompensated schistosomiasis cases, HF may hold back the liver portal system, causing severe portal hypertension with bleeding esophageal varices and end-stage liver failure. Unlike other liver injuries that could directly lead to hepatocellular damage (such as viral hepatitis), the schistosomiasis-induced lesion is localized to the vascular endothelium. Previous case-control studies have examined the polymorphisms of specific gene-encoded proteins regulating inflammation and fibrosis. In the present study, we scanned the whole genome to find more susceptibility genes. All samples were collected from schistosome-infected patients, which may avoid the type II error (false-negative error). Because uninfected subjects from the general population are still at risk of developing severe schistosomiasis, the inclusion of such a control group limits the ability to obtain positive results when comparing contributions of SNPs in control subjects with those in patients with severe schistosomiasis. In addition, we could not completely divide all patients into chronic and advanced categories according to the diagnostic criteria for schistosomiasis. For example, many patients had severe liver injury without ascites and abnormal PVD. Therefore, we conducted GWAS analyses on original indicator data. Likewise, we did not combine biochemical indicators of liver injury (HA, AST, albumin) but used their raw measurements, thus reducing the loss of useful information. We will report in the next article for the susceptibility gene study of schistosomiasis HF.

However, there were several limitations to our analysis. First, we did not replicate the candidate loci in the other independent schistosomiasis cohort. This is due to the impact of the COVID-19 pandemic on the investigation and collection of case samples. Although through eQTL association and SMR analyses, we identified partial causal genes (*ITIH4*, *PMEPA1*, *MAMSTR*) for schistosomiasis complications, some genes (*RP11-61O1.1*, *OGDHL*) did not show evidence of causality, which is indeed a limitation of this study. Therefore, further experimental investigation is required to elucidate the function and mechanism of candidate genes.

?A3B2 tlsb-.1pt?>In conclusion, our research highlights six novel loci conferring susceptibility to severe schistosomiasis complications. Six candidate genes were upregulated in the livers of schistosomiasis-infected mice, suggesting their involvement in schistosomiasis development. Our analyses also indicated that *ITIH4*, *PMEPA1*, and *MAMSTR* are potentially causal genes affecting the severity of schistosomiasis. The identification of genetic factors in hosts might provide novel insights into disease susceptibility, ultimate outcomes and treatments. Our results provide several lines of evidence supporting candidate genes that could serve as potential targets of precise prevention and treatment strategies. This could improve the survival rate and quality of life for patients with advanced schistosomiasis.

## Data Availability Statement

The datasets presented in this study can be found in online repositories. The names of the repository/repositories and accession number(s) can be found below: The GWAS summary datasets generated for this study can be found here https://pmglab.top/resource/gwas/sj.

## Ethics Statement

The studies involving human participants were reviewed and approved by Ethics Committees of Fudan University (IRB#2016-TYSQ-03-17). The patients/participants provided their written informed consent to participate in this study. The animal study was reviewed and approved by Human Research Ethics Committee of the Sun Yat-Sen University (No·2016-104).

## Author Contributions

Conceptualization, MZ, CX, ZW, XW, and ML. Data curation, MZ and XW. Formal analysis, MZ and CX. Investigation, MZ, CX, ZW, XW, and ML. Methodology: MZ and CX. Visualization, MZ and CX. Writing - original draft, MZ. Writing - review and editing, all authors. Supervision, XW and ML. Funding acquisition, ML. All authors contributed to the article and approved the submitted version.

## Funding

This research was funded by Science and Technology Program of Guangzhou, grant number 201803010116. Guangdong project, grant number 2017GC010644 and National Natural Science Foundation of China, grant number 32170637.

## Conflict of Interest

The authors declare that the research was conducted in the absence of any commercial or financial relationships that could be construed as a potential conflict of interest.

## Publisher’s Note

All claims expressed in this article are solely those of the authors and do not necessarily represent those of their affiliated organizations, or those of the publisher, the editors and the reviewers. Any product that may be evaluated in this article, or claim that may be made by its manufacturer, is not guaranteed or endorsed by the publisher.
