# Supplementary material for: Genome-Wide Association Study Identifies New Risk Loci for Progression of Schistosomiasis Among the Chinese Population
Source: Front Cell Infect Microbiol. 2022 Apr 12;12:871545. doi: 10.3389/fcimb.2022.871545 (PMC9039613; doi:10.3389/fcimb.2022.871545)
Supplement: Supplementary file 1 [file Presentation_1.pptx]

## Slide 1
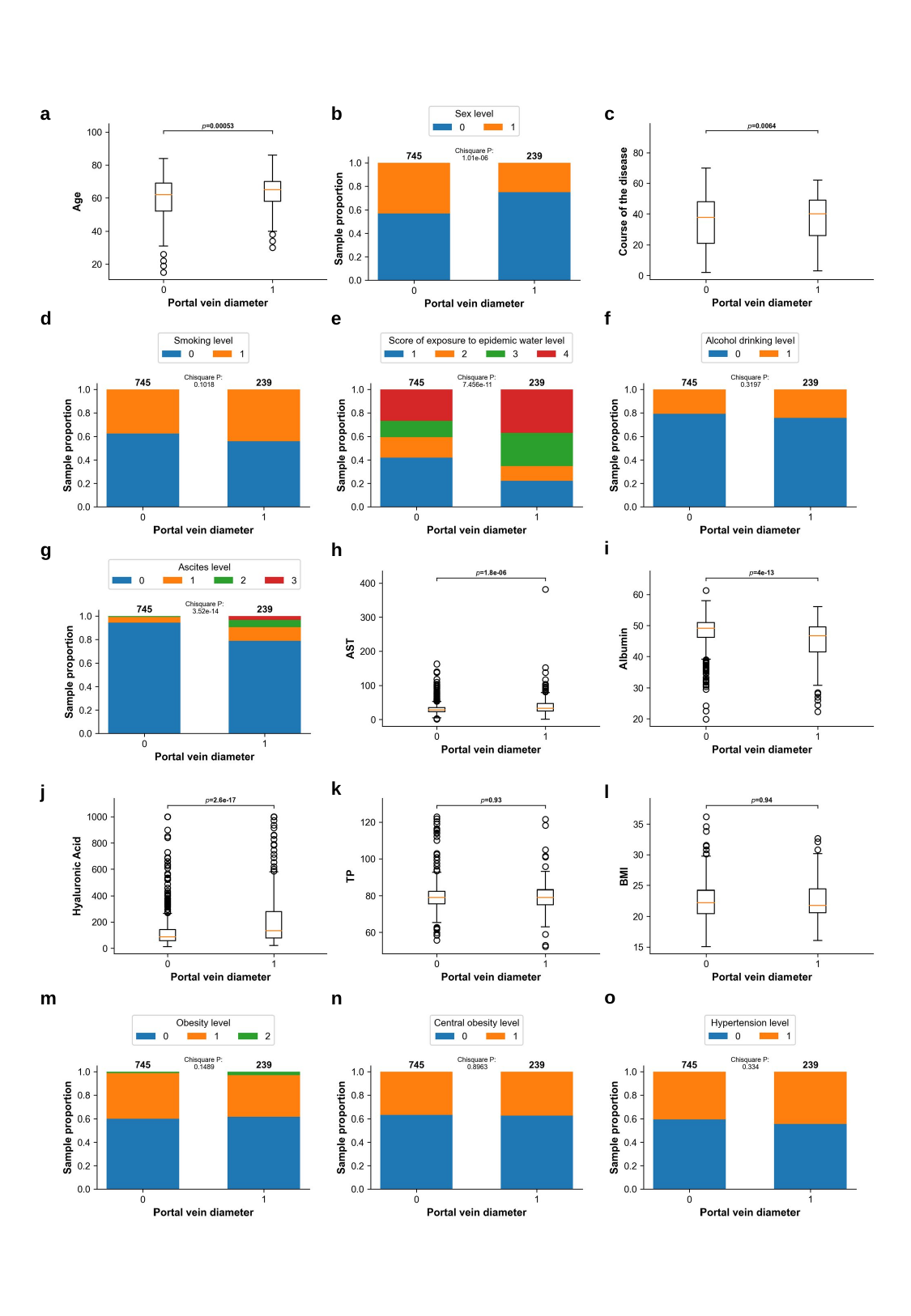

a
b
c
d
e
f
i
g
h
k
j
l
o
n
m

## Slide 2
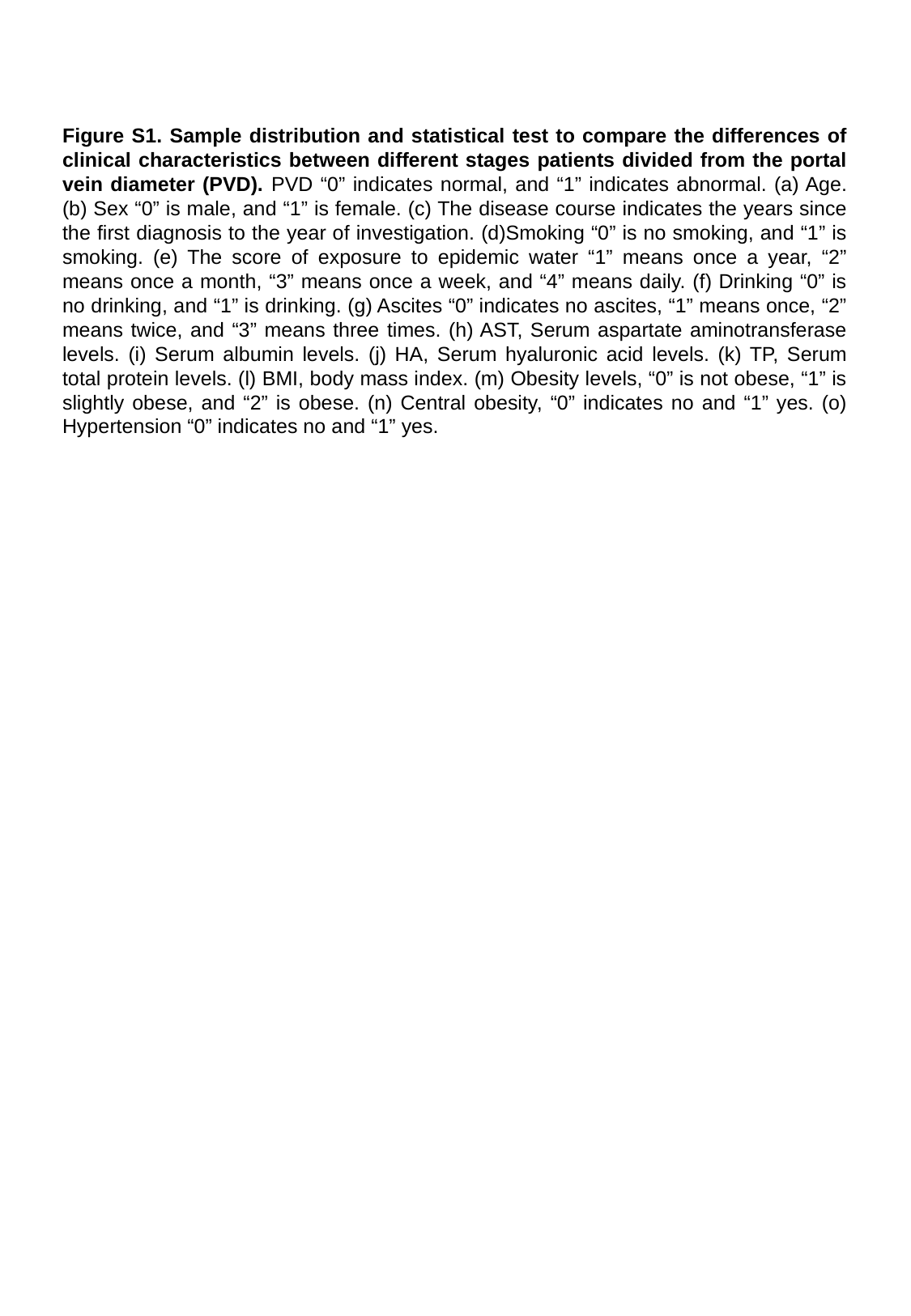

Figure S1. Sample distribution and statistical test to compare the differences of clinical characteristics between different stages patients divided from the portal vein diameter (PVD). PVD “0” indicates normal, and “1” indicates abnormal. (a) Age. (b) Sex “0” is male, and “1” is female. (c) The disease course indicates the years since the first diagnosis to the year of investigation. (d)Smoking “0” is no smoking, and “1” is smoking. (e) The score of exposure to epidemic water “1” means once a year, “2” means once a month, “3” means once a week, and “4” means daily. (f) Drinking “0” is no drinking, and “1” is drinking. (g) Ascites “0” indicates no ascites, “1” means once, “2” means twice, and “3” means three times. (h) AST, Serum aspartate aminotransferase levels. (i) Serum albumin levels. (j) HA, Serum hyaluronic acid levels. (k) TP, Serum total protein levels. (l) BMI, body mass index. (m) Obesity levels, “0” is not obese, “1” is slightly obese, and “2” is obese. (n) Central obesity, “0” indicates no and “1” yes. (o) Hypertension “0” indicates no and “1” yes.

## Slide 3
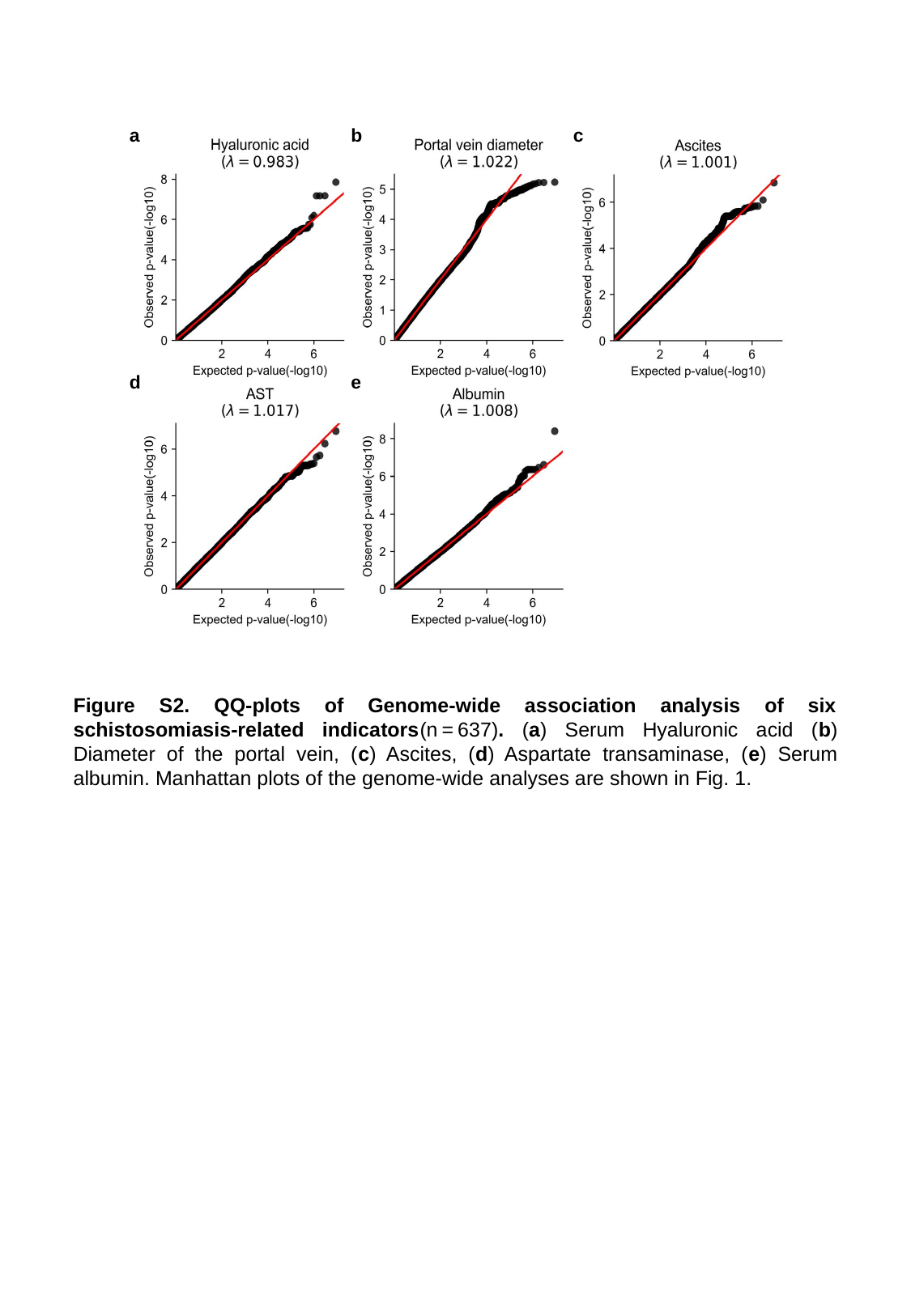

a
b
c
d
e
Figure S2. QQ-plots of Genome-wide association analysis of six schistosomiasis-related indicators(n = 637). (a) Serum Hyaluronic acid (b) Diameter of the portal vein, (c) Ascites, (d) Aspartate transaminase, (e) Serum albumin. Manhattan plots of the genome-wide analyses are shown in Fig. 1.

## Slide 4
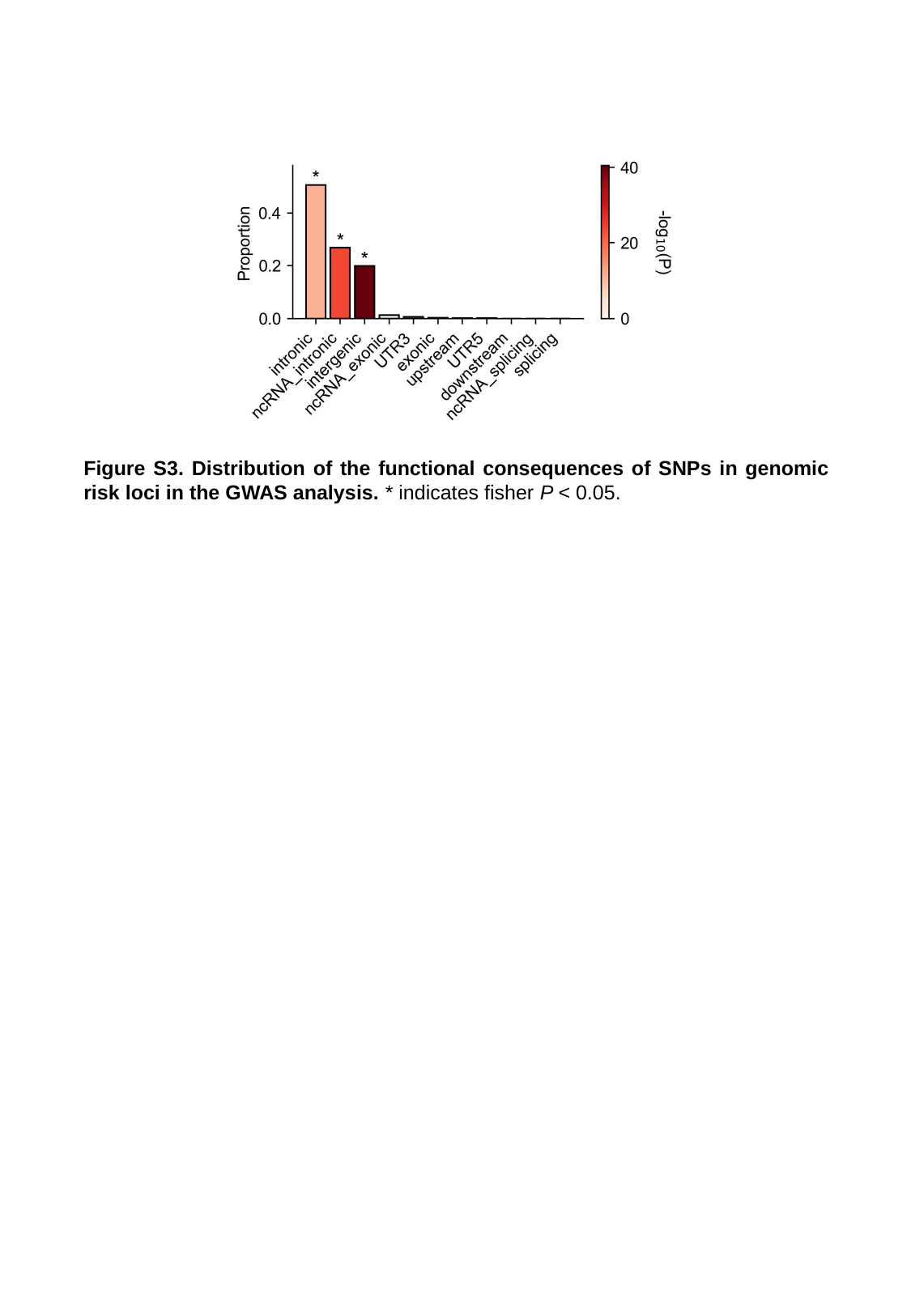

Figure S3. Distribution of the functional consequences of SNPs in genomic risk loci in the GWAS analysis. * indicates fisher P < 0.05.

## Slide 5
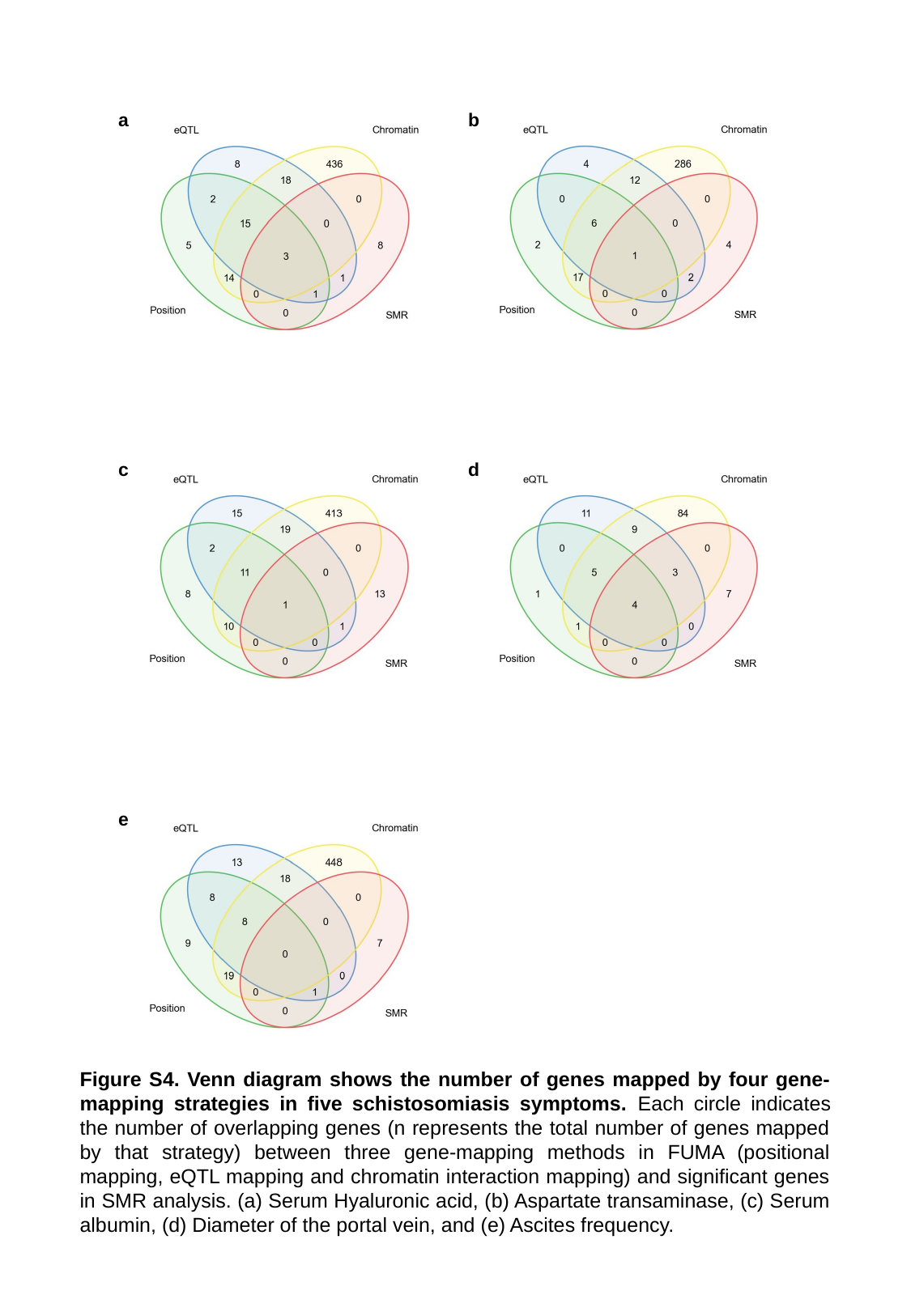

a
b
c
d
e
Figure S4. Venn diagram shows the number of genes mapped by four gene-mapping strategies in five schistosomiasis symptoms. Each circle indicates the number of overlapping genes (n represents the total number of genes mapped by that strategy) between three gene-mapping methods in FUMA (positional mapping, eQTL mapping and chromatin interaction mapping) and significant genes in SMR analysis. (a) Serum Hyaluronic acid, (b) Aspartate transaminase, (c) Serum albumin, (d) Diameter of the portal vein, and (e) Ascites frequency.

## Slide 6
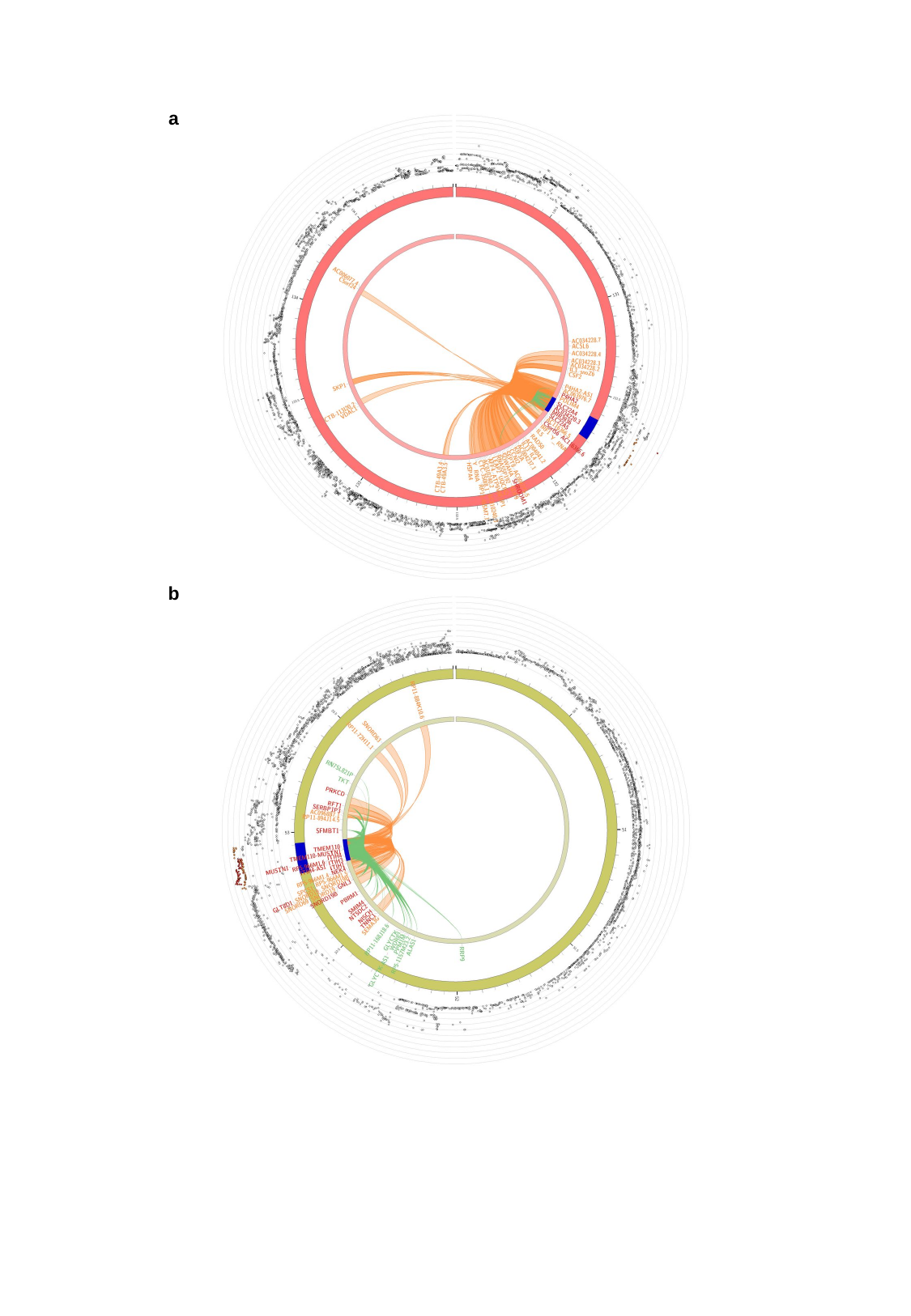

a
b

## Slide 7
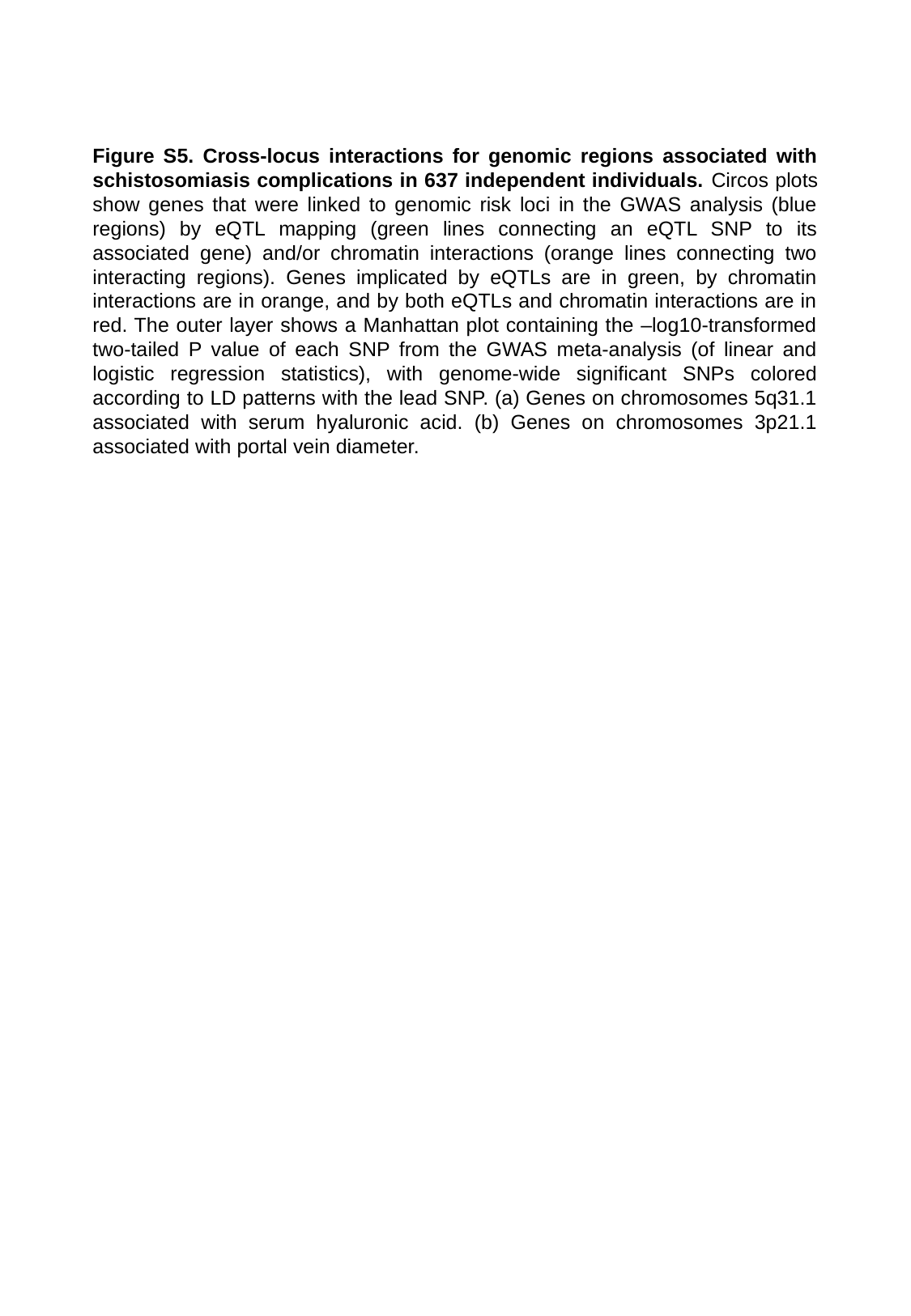

Figure S5. Cross-locus interactions for genomic regions associated with schistosomiasis complications in 637 independent individuals. Circos plots show genes that were linked to genomic risk loci in the GWAS analysis (blue regions) by eQTL mapping (green lines connecting an eQTL SNP to its associated gene) and/or chromatin interactions (orange lines connecting two interacting regions). Genes implicated by eQTLs are in green, by chromatin interactions are in orange, and by both eQTLs and chromatin interactions are in red. The outer layer shows a Manhattan plot containing the –log10-transformed two-tailed P value of each SNP from the GWAS meta-analysis (of linear and logistic regression statistics), with genome-wide significant SNPs colored according to LD patterns with the lead SNP. (a) Genes on chromosomes 5q31.1 associated with serum hyaluronic acid. (b) Genes on chromosomes 3p21.1 associated with portal vein diameter.

## Slide 8
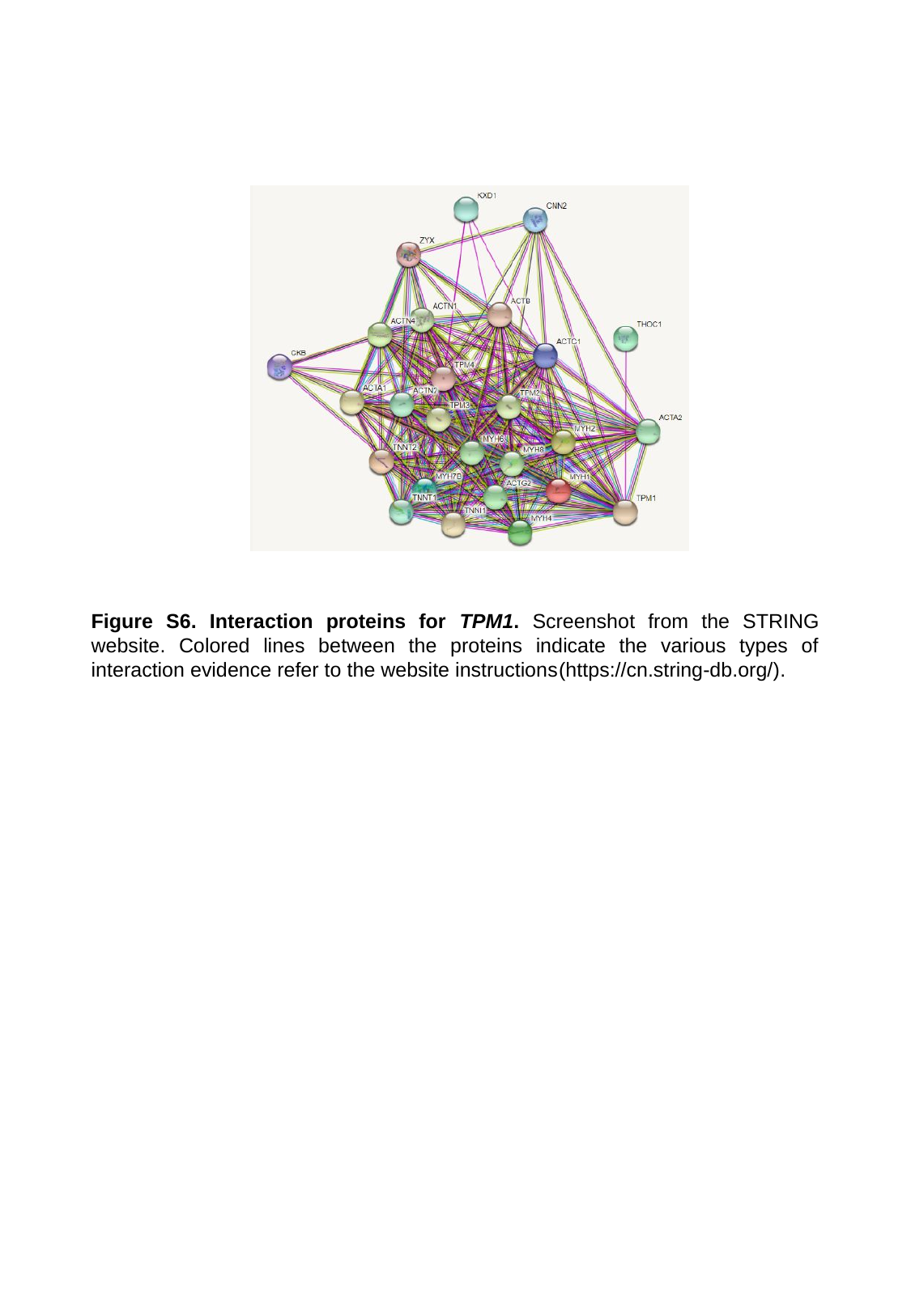

Figure S6. Interaction proteins for TPM1. Screenshot from the STRING website. Colored lines between the proteins indicate the various types of interaction evidence refer to the website instructions(https://cn.string-db.org/).

## Slide 9
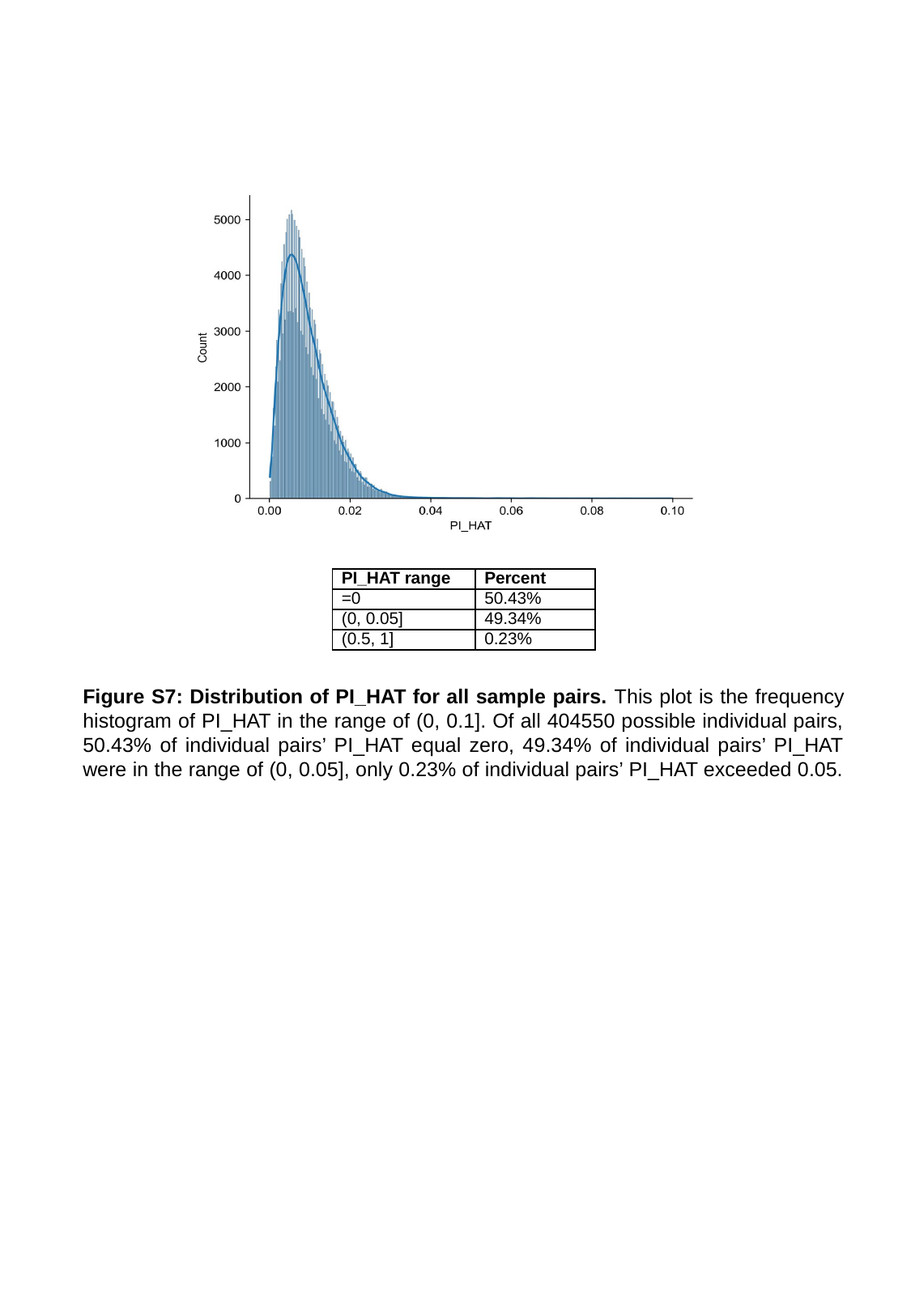

| PI\_HAT range | Percent |
| --- | --- |
| =0 | 50.43% |
| (0, 0.05] | 49.34% |
| (0.5, 1] | 0.23% |
Figure S7: Distribution of PI_HAT for all sample pairs. This plot is the frequency histogram of PI_HAT in the range of (0, 0.1]. Of all 404550 possible individual pairs, 50.43% of individual pairs’ PI_HAT equal zero, 49.34% of individual pairs’ PI_HAT were in the range of (0, 0.05], only 0.23% of individual pairs’ PI_HAT exceeded 0.05.
